# On Modern Progress in Ophthalmic Medicine and Surgery

**Published:** 1895-03-16

**Authors:** Robert Brudenell Carter

**Affiliations:** Consulting Ophthalmic Surgeon to St. George's Hospital


### On Modern Progress in Ophthalmic Medicine and Surgery.

By Robert Brudenell Carter, F.R.C.S., Consulting Ophthalmic Surgeon to St. George's Hospital.

### DISEASES OF THE CORNEA.

(*Continued from page 343.*)

The cornea, like most other textures of the body, is liable to undergo ulceration, which may be limited to its epithelial covering, or may extend more deeply into the true tissue of the membrane. In the latter case, even in the most favourable circumstances, an ulcer will leave a visible cicatrix, which may vary from the slightest cloud upon the transparency of the surface to a dense patch of white opacity, which, if it should occupy the pupillary area, must of necessity be an impediment to vision. Moreover, the healing process is apt to be attended by gradual contraction of the cicatrix, by which, in an ulcer of any but the smallest dimensions, the curves of the unaffected portions of the cornea are changed in the direction of flattening, sometimes to so great a degree that vision is much impaired, even through the portions which still remain transparent. On both these grounds it is of the first importance to arrest the ulcerative process at the earliest possible stage, and to limit alike its superficial extension and its extension in the direction of depth.

Ulceration of the cornea is seen under three very definite types. There are ulcers which depend upon the presence of pathogenic microbes within the body; others which depend upon faulty nutrition of the tissue, due, presumably, to some defect in its innervation; and others which are produced by injury, and afterwards infected by microbes derived from the atmosphere, or from the secretions of the conjunctiva.

Ulcers produced by pathogenic microbes within the body are of two kinds, the so-called phlyctenulæ, which are usually localised manifestations of tuberculosis, and the ulcers left by the breaking up of the tissue in front of a corneal abscess.

Phlyctenulæ are usually multiple and recurrent. They chiefly occur in strumous children, and they commence as small red pimples or elevations, either on the very margin of the cornea or just within the margin, although they are sometimes seated on the ocular conjunctiva. Each successive pimple ulcerates at its apex, exposing a grey or yellowish slough, which is cast off in a few days, leaving a healing surface. The advancing stage is attended by general flushing of the conjunctiva, with more or less smarting and intolerance of light, the latter symptom being sometimes very distressing and severe. Whether single or multiple, the pimples are apt to appear in successive crops over a long period of time. Each one, when seated on the true cornea, leaves at the best a faint nebula behind it; and, in unfavourable or neglected cases, these nebulæ may be definitely flattened facets, and may even be to some extent causes of visual disturbance, although this is comparatively rare.

The remedies which are now known to be active bactericides were found, long before the influence of pathogenic microbes was even suspected, to be highly valuable for the cure of phlyctenulæ; and, among these remedies, preparations of mercury hold the first place. Such preparations are most effective when used in the form of powders or ointments, because these remain in contact with the diseased tissue longer than a solution. Dusting the eye with dry calomel was at one time a favourite practice; but it is open to the objection that, if the patient be at the same time taking iodide of potassium internally, it may occasion a formation of iodide of mercury within the conjunctival sac, and thus produce great pain and irritation or even permanent injury. The red nitric-oxide, finely powdered and made into an ointment, has also been used; but, some thirty years ago, the late Dr. Pagenstecher called attention to the advantages of the yellow oxide, the basis of the well-known "yellow wash," and it has since almost superseded other preparations. Being prepared by precipitation, it is an impalpable powder, and produces its chemical or medicinal effects without acting in any degree as a mechanical irritant. It is made into an ointment with vaseline or other inert fat, and about a grain to a drachm is usually the best strength. The ointment may be conveniently sent out in tubes, like those employed for colours by artists, and from such a tube a piece of ointment as large as a hempseed may be squeezed into the lower conjunctival sac twice a day, and distributed by gentle friction over the closed lids. The ulcers left by phlyctenulæ will rapidly heal under its influence.

The whole history of a case of phlyctenulæ shows, however, that the pathogenic microbes are present in the meshes of the sub-conjunctival tissue, and that they travel from thence towards the cornea, or actually into its texture. Sometimes, as already mentioned, they produce their special effects on the conjunctiva; but, as a rule, they are innocuous until the cornea has been reached. Modern knowledge of the true character of the morbid process has pointed to a new method of controlling it, and to a way of using mercury as a bactericide more effectual than any which had previously been suggested. This way is by sub-conjunctival injection. For this purpose, a very young child may have a little chloroform to prevent struggling; but, with a child of ten or thereabouts, it will usually be enough to cocainise the affected eye thoroughly. A fold of conjunctiva should then be pinched up with forceps, and a few drops of a solution of mercuric perchloride, of 1 in 4,000, should be gently injected by an ordinary hypodermic syringe. The liquid will find its way to every part of the sub-conjunctival tissue, and a single dose will often be sufficient to put a complete stop to the malady. Any ulcers which are unhealed at the time will require no other treatment than such protection as may be given by closure of the lids for a short period, by means of a light pad of cotton wool and some form of retaining band.

The excessive intolerance of light with which phlyctenulæ are sometimes associated appears to depend upon the exposure of a nerve filament in the cavity of an ulcer, and is often aggravated by the forcible closure of the lids as a means of excluding light. Such closure retains tears and other irritating matters, and exerts an injurious pressure upon the tender surface of the eye. The photophobia will usually yield at once to cocaine and mercurial injection; but, in very obstinate cases, it is occasionally necessary to relax the pressure by division of the orbicularis muscle at the external canthus.

